# Supplementary material for: MetaRibo-Seq measures translation in microbiomes
Source: Nat Commun. 2020 Jun 29;11:3268. doi: 10.1038/s41467-020-17081-z (PMC7324362; doi:10.1038/s41467-020-17081-z)
Supplement: Supplementary file 10 — Supplementary Data 7 [file 41467_2020_17081_MOESM10_ESM.zip › File2/Confidence_High_Taxonomy/141215_out.krona.html]

Javascript must be enabled to view this page.

members
magnitude
magnitudeUnassigned
count
unassigned
taxon
rank

141215\_out

2

2
superkingdom
2

1
976
phylum

class
117743
1

order
200644
1

49546
family
1

genus
1016
1

species
1019
1

SRS049318\_contig\_number\_21725

1
1239
phylum

1
186801
class

1
186802
order

31979
family
1

1
1485
genus

species
1262800
1

SRS024132\_contig\_number\_11005
